# A View to the Future: Opportunities and Challenges for Food and Nutrition Sustainability

**DOI:** 10.1093/cdn/nzaa035

**Published:** 2020-03-18

**Authors:** Eileen Kennedy, Daniel Raiten, John Finley

**Affiliations:** 1 Friedman School Nutrition Science and Policy, Tufts University, Boston, MA, USA; 2 National Institutes of Health, Bethesda, MD, USA; 3 Agricultural Research Service, USDA, Beltsville, MD, USA

**Keywords:** diet, nutrition, sustainability, Sustainable Development Goals, agriculture, Front-of-Pack, environment

## Abstract

The challenges to achieving sustainability in food and nutrition are daunting. The present paper summarizes 3 individual papers that are part of this special collection. The lynchpin for synthesizing the papers is sustainability and food systems. Within each of these domains are embedded a myriad of factors, each of which are essential for the sustainable transformation of food systems. Controversies surrounding the concepts of a healthy diet, sustainable agricultural production, and maximizing the dietary impacts of food environments are discussed and evaluated in the context of the current food and nutrition landscape.

## Teaser

Food systems globally are changing rapidly. As food systems evolve, there have been burgeoning rates of overweight and obesity. There is now a clarion call for transformational changes in food systems, worldwide, that enhance diet quality and improve nutritional status. The challenges is daunting. Food Systems are expected to improve both under and over nutrition, and to do so in a manner that is respectful of natural resources. This paper assesses challenges for developing sustainable food systems and implications for designing effective strategies.

## Introduction

The 17 UN Sustainable Development Goals (SDGs) provide a blueprint for sustainable global development for current and future generations (1). SDG-2 focuses on food security and nutrition, specifically targeting zero hunger, achieving food security, eliminating malnutrition in all its forms, and promoting sustainable agriculture. SDG-12, a related goal, calls for responsible production and consumption. Our ability to achieve these goals will demand on some introspection with regard to the current and future food production, global nutrition, and health.

Advances in agriculture, including biotechnology, have resulted in major gains in food security, nutrition, poverty alleviation, employment, and overall development (2). Since 1945, total food production has tripled and the average caloric availability has risen by 40% (3). These advances, by what is commonly called the Green Revolution, have resulted in increased food availability and important public health gains, such as a significant decrease in protein/calorie malnutrition (4).

However, these successes have come at a cost to agricultural resources and the environment. They have negatively impacted water and land resources and substantially contributed to greenhouse gas (GHG) emissions (5). Many public health problems have actually increased as evidenced by dramatic increases in obesity and related comorbidities (6); moreover, micronutrient malnutrition still plagues a substantial portion of the earth's population (7). The challenge going forward is to launch a “greener¸ green revolution” that achieves improved agricultural production and public health while respecting natural resources.

Agriculture, public health, and the environment are deeply interconnected and achieving successes in all areas will require a new paradigm of open, frank, yet collegial, cross-disciplinary discourse and interaction that is not simply prescriptive (8). It will require definitions of boundaries and metrics used to measure success/failure. Discussions of responsible production and consumption cannot be assessed piecemeal and particularly require assessment of tradeoffs between sustainable production and healthy diets. The focus and scope of this special collection are designed to support these important conversations.

## Consensus Objectives

The Climate/environmental change, Health, Agriculture, Improving Nutrition (CHAIN) Research Interest Group of the ASN focuses on research, knowledge, and capacity development to support sustainable food systems, health, and nutrition in a changing global environment. CHAIN sponsored 3 sessions at ASN 2019 (9–11). While the specific objectives of the individual sessions varied somewhat, there were 2 overarching themes that linked the sessions: *1*) sustainability and *2*) food systems. This paper is a summary of some of the key messages that emerged from the 3 CHAIN-sponsored sessions.

## Nutrients, Foods, Diets, People: Promoting Healthy Eating

Positive growth in agricultural production does not guarantee a healthy, sustainable diet. The EAT Lancet planetary health reference diet discussed in this collection (12) provides clear recommendations for nutritional adequacy. The reference diet, however, presents serious dilemmas for widespread adoption and the increases in agricultural production that will be required are challenging. The feasibility of these production changes needs to be considered within the context of current national agricultural systems and how and if these strategies will or can be adapted to achieve agricultural production targets. For example, the reference diet would require a >150% increase in nut production (12). It should be noted that the development of the planetary health diet is based on nutrition considerations, not environmental factors.

The challenges to agriculture are daunting; agriculture will need to meet the food needs of a growing population, for the most part, on the same amount of land and with a declining labor base (13). Agriculture will be expected to continue to reduce malnutrition by increasing food availability while simultaneously improving food access to keep pace with population growth. Successful, sustainable agricultural strategies will need to increase incomes, particularly for the rural poor, be a major vehicle for employment generation, improve agriculture output, preserve natural resources, and contribute to food security and diets.

## Micronutrient Nutrition and Animal-Source Foods

As an omnivore, animal-source foods (ASFs) have been a central component of the human diet throughout evolution. In addition to high-quality protein, ASFs are a rich source of micronutrients, and micronutrient deficiency is a persistent problem across all cultures. In Western cultures, iron deficiency is ubiquitous in some subgroups (14), and in the global south, poor quality, primarily carbohydrate diets have resulted in severe micronutrient insufficiency (e.g., iron, zinc, vitamin A, or vitamin B-12) (15). The context-specific options for improving micronutrient status and improving overall diet quality will need to include a role for ASFs (10).

Because ASFs are a rich source of high-quality protein and a range of micronutrients, there are tradeoffs between sustainable agricultural techniques and the amount of ASFs in dietary patterns. While the potential contribution of ASFs in meeting nutritional needs is incontrovertible, controversies about the role of ASFs include implications for health, social/cultural practice, and sustainability. With specific regard to the latter, the primary issue is around the relative contribution of animal agriculture to GHG emissions, primarily methane. The relative net impact of ASFs in comparison to the nutritional output is not settled science and is complicated by our understanding of net GHG accumulation, resource depletion, and the tradeoff of efficiently meeting nutritional needs while exploiting land resources that would otherwise not be available for food production. Fully understanding the impacts of food production systems such as ASFs requires integrated modeling techniques such as Life Cycle Analysis.

Our ability to provide answers to these questions will have a significant impact on decisions about not only the role of ASFs in meeting nutritional needs domestically and globally but also the sustainability of agricultural enterprises in a changing environment.

## Transforming Food Systems

The UN has specified that sustainable food systems are critical for promoting healthy diets (16). Food systems gather all the elements (environment, people, inputs, processes, infrastructure, institutions) and activities related to the production, processing, distribution, preparation, and consumption of food (17); a sustainable food system takes this definition further to include food systems that ensure food security and nutrition for all, without compromising the socioeconomic, environmental, and social bases for current and future generations (17). An FAO report has noted, “The way in which agriculture and food systems develop over the next 15 years is key to success in reaching the SDGs” (18). The challenge for international organizations and national-level governments is to change the trajectories of food systems to maximize the food security and nutrition impacts. This will be a mammoth task given the amount of malnutrition; in addition, by 2050, food systems will need to feed >9 billion and demand for livestock will grow by 70%, with much of this increase occurring in developing countries (19).

A holistic approach is needed to address challenges of sustainability, environmental degradation, persistent poverty, vulnerability, and hunger and malnutrition. However, the opportunities to respond are enormous, and a new, collective, and integrated approach is imperative. Food systems need to be more efficient and inclusive and the policies and legal frameworks around food systems should address income inequality, supporting livelihoods, and ensuring resilience, while ensuring coherent and effective national and international governance.

Food system theory is well documented and, recently, illustrative conceptual frameworks of these theories have been reported (17); however, there are few examples of where the impacts of the entire food systems have been tested. There are many food system drivers suggestive of a range of positive and negative impacts across the production to consumption spectrum. One way of evaluating these effects is to decompose them into the 4 domains and basic pillars of health, economics, environment, and society (9).

The challenge for sustainable diets is to balance nutrient requirements, costs, and cultural acceptance within environmental and societal norms. The papers in this series highlight the fact that cost of food is usually a limiting constraint to accessing a healthy diet (9, 11). Nutrient-rich foods cost more (9); conversely, nutrient-poor foods and diets are cheaper and thus more likely to be consumed (9).

## Discussion

The challenges for achieving the targets set forth for SDG-2 and SDG-12 have been raised in the previous sections; however, it is clear that these challenges are interconnected and cannot be addressed in isolation. Policies addressing priorities will be needed, but the prevailing model of governance assigns malnutrition to the health sector and food insecurity to agriculture, resulting in a disjointed and uncoordinated framework, which may work at cross-purposes. Choices must be made by the consumer, producer, and those developing policy. Overcoming these challenges, informing policy, and developing a system that promotes optimal health, environmental and economic sustainability, and appeals to consumer choice will require an integrated framework.

Progress has been made over the past 50 y in improving food security, nutrition, and income. Further progress will require concerted efforts to ensure that food security and nutrition continue to be a priority in the development agenda.

## Notes

The authors reported no funding received for this study.

Author disclosures: The authors report no conflicts of interest. EK and JF are Editors of *Current Developments in Nutrition*; they were not involved in the peer review of this article.

Abbreviations used: ASF, animal-source food; CHAIN, Climate/environmental change, Health, Agriculture, Improving Nutrition; GHG, greenhouse gas; SDG, Sustainable Development Goal.
